# A study of the mechanisms of Anaphylaxis through passive transfer of IgE-mediated cutaneous reactivity

**DOI:** 10.1186/2045-7022-5-S1-O9

**Published:** 2015-03-11

**Authors:** Anja Pahlow Mose, Charlotte Gotthard Mørtz, Esben Eller, Signe Inglev Steffansen, Carsten Bindslev-Jensen

**Affiliations:** 1 Odense University Hospital, Department of Dermatology and Allergy Centre, Odense, Denmark; 2 Odense University Hospital, Department of Dermatology and Allergy Centre, Odense; Denmark; 3 Odense University Hospital, Department of Nuclear Medicine, Odense, Denmark

## Background

A large number of questions regarding the mechanisms of anaphylaxis remain unanswered. One such question is the following: what determines the rate at which the allergic reaction propagates from the point of entry of the allergen in the mouth to full-blown systemic response? We present here a study based on the principle of the original experiments by Prausnitz and Küstner. The aim was to study the importance of different serum profiles of patients with peanut allergy on the dissemination of the allergic reaction.

## Method

Sera from patients with allergy to peanut and with different S-IgE profiles to peanut and Ara h2 were collected at our outpatient clinic. After appropriate testing and approval for safety reasons, the sera were used for passive transfer of peanut-IgE by intradermal injection of the undiluted or diluted sera into the volar forearms of non-allergic subjects. 24 hours following priming, the subjects underwent oral challenges (“single-shot” or titrated) with roasted peanut.

## Results

A positive wheal and flare reaction first appeared at the primed skin site of the undiluted serum with the highest IgE to Ara h2 (T_mean_= 26 min). Subsequently, the reaction spread to the primed skin sites of higher serum dilutions in a dose-response-like manner. Positive reactions developed more quickly in skin sites primed with the same undiluted serum when subjects were given a higher dose of peanut (10 g) compared to a smaller dose (0,3 g). Interestingly, some subjects required a larger dose of peanut to elicit a positive reaction (threshold) even though the subjects had been primed using the same undiluted serum.

## Conclusion

Based on these results, several factors such as the quantity and “quality” of S-IgE of peanut appears to play a significant role in the rate at which the allergic reaction disseminates to the end-organs. Other factors involved remains to be elucidated.

